# Proposing the potential of utilizing the CAT score for early detection of COPD in asymptomatic patients, shifting towards a patient-centered approach: A review

**DOI:** 10.1097/MD.0000000000037715

**Published:** 2024-04-12

**Authors:** Atefeh Vaezi, Mehdi Mirsaeidi

**Affiliations:** a Division of Pulmonary, Critical Care, and Sleep Medicine, College of Medicine-Jacksonville, University of Florida, Jacksonville, FL; b Division of Pulmonary, Critical Care, and Sleep Medicine, College of Medicine, University of Florida, Jacksonville, FL.

**Keywords:** CAT score, chronic obstructive, COPD assessment tool, diagnostic screening programs, pulmonary disease

## Abstract

Chronic obstructive pulmonary disease (COPD) constitutes a significant public health challenge, with delayed diagnosis and underdiagnosis being pervasive issues. The United States Preventive Service Task Force recommends restricting COPD screening to symptomatic smokers, a focus that has exhibited limitations, leading to delayed diagnoses, and imposing a substantial burden on patients, their families, and the healthcare system. This paper explores an alternative approach, highlighting the potential utility of the COPD assessment test (CAT) score as a prescreening tool. A CAT score of 10 or higher could serve as an appropriate threshold for further diagnostic procedures, given its robust correlation with pulmonary function test parameters and is valuable capacity to quantify patients’ symptoms. The utilization of CAT as a prescreening tool in primary care signifies a transition towards a more patient-centered and comprehensive approach to COPD diagnosis and care.

## 1. Introduction

The United States Preventive Services Task Force (USPSTF) recommended against screening for chronic obstructive pulmonary disease (COPD) in asymptomatic adults in 2016 and reiterated the same recommendation in 2022.^[1]^ COPD has emerged as a significant and pressing public health concern, advancing from being the 6th leading cause of mortality in 1990 to currently holding the third position.^[2]^ The global prevalence of COPD was approximately 10% in 2019, encompassing nearly 390 million individuals.^[3]^

The 2006 BOLD study (burden of obstructive lung disease) discovered a worldwide prevalence of stage II or higher COPD at 10%, implying a higher prevalence of advanced stages.^[4]^ The Latin American Project for the Investigation of Obstructive Lung Disease, launched in 2002 across 5 Latin American countries, reported varying COPD rates ranging from 7.8% to 19.7%. these rates were higher in men, older adults, less educated individuals, and those with greater exposure to smoking.^[5]^ In the United States, the overall age-adjusted prevalence of COPD surveyed by the Behavioral Risk Factor Surveillance System was 6.2% in 2017.^[6]^ Given the increasing prevalence of smoking, heightened exposure to dust and air pollution due to global climate change, and an aging population, the rising incidence of COPD remains at the forefront of concern.^[2]^

The impact of COPD, whether diagnosed or undiagnosed, extends beyond health implications and encompasses substantial economic burdens. Patients with COPD lost an average of 4.6 workdays per 6 months.^[7]^ In the Unites States, the combined national healthcare cost, along with expenses related to absenteeism attributed to COPD, totaled nearly $36 billion in 2010.^[8]^ Projections indicated that these costs will increase to $57.86 billion for direct costs and $6.97 billion for indirect cost in 2038.^[9]^ When compared to other chronic diseases, patients with COPD experience approximately double the healthcare costs incurred by other patient populations.^[10]^

The national and international COPD guidelines, including the USPSTF, have emphasized the presence of clinical symptoms in addition to spirometry evidence of persistent bronchial obstructive defect for COPD diagnosis. According to the global initiative for chronic obstructive lung disease (GOLD), COPD is diagnosed with persistent respiratory symptoms along with a reduced forced expiratory volume in 1 second (FEV1) to forced vital capacity ratio of under 0.70 in spirometry.^[11]^ However, none of these organizations have explored the limitation of subjectivity of clinical symptoms in a chronic respiratory disease and the risk of misleading the screening efforts.

Considering the significant gap in the diagnosis of COPD and the undeniable social and economic consequences it entails, this prospective article aims to raise concerns about the unreliability of subjective findings in COPD guidelines, especially the current USPSTF recommendations. The article advocates for a more compassionate screening approach for COPD in the United States. By presenting compelling evidence and a comprehensive analysis, we propose a revised screening recommendation that emphasizes the importance of early detection and prompt intervention in mitigating the impact of COPD on public health and overall well-being.

## 2. COPD symptoms and adaptation

### 2.1. The spectrum of COPD symptoms

Dyspnea, cough, and sputum are the most prevalent symptoms reported by patients with COPD with severe airflow obstruction. Among current smokers, cough and sputum are prevailing symptoms, while among former smokers, dyspnea is the predominant symptom.^[12,13]^ Approximately 45% of patients with COPD report moderate to severe dyspnea, which is defined as an effort to breathe.^[14]^ The overall prevalence of frequent cough with and without sputum in patients with COPD is around 40% and 60%, respectively.^[15,16]^ Apart from cough, the production of sputum is another symptom that occurs mostly in those with chronic bronchitis phenotype. Changes in the quantity of sputum or its becoming purulent are associated with an upcoming exacerbation,^[17]^ which is considered a contributor to COPD-related mortality.

### 2.2. Patient adaptation and quality of life

The emphasis on being symptomatic in COPD screening has probably been overstated. The concept of “not truly asymptomatic” versus “truly asymptomatic” patient in COPD was proposed by Yawn and colleagues.^[18]^ In COPD, “fake” asymptomatic patients are those who do not consider their symptoms as “real” symptoms, instead attributing them to daily on-and-off issues. For instance, it has been seen that the objective level of dyspnea is not correlated with patients’ perception of their disease severity. Around one-third of patients with COPD who suffer from severe dyspnea perceive their condition as mild to moderate.^[19]^ A gradual reduction in lung function does not necessarily prompt individuals to consult a physician; instead, they are more likely to visit their physician when they experience fluctuations in respiratory symptoms or a decline in their quality of life.^[20,21]^

Respiratory symptoms stay subjective until diagnostic tests are conducted. It is inherent in human nature to adapt to various challenges. Consequently, respiratory symptoms alone may not provide reliable measures for assessing airway obstruction. Patients with COPD, whether diagnosed or undiagnosed, often adjust their activity levels to avoid reaching their tolerance limits and experiencing dyspnea. Most of them face restrictions in various aspects of their lives, including daily activities, family-related engagements, social interactions, sleep patterns, work abilities, and participating in regular physical activities.^[22]^

Van Schaych and his team have demonstrated that the perception of symptoms plays a crucial role in predicting whether patients with asthma seek medical attention. Given the gradual progression of COPD, it is crucial to recognize that not all airway obstructions result in bronchial symptoms, making under-presentation to healthcare providers an even more significant concern compared to patients with asthma. As the disease may develop gradually, some individuals with COPD might not experience noticeable respiratory symptoms until the condition has significantly advanced.^[23]^ Patients with COPD who experience symptoms may be at higher stages of the disease’s severity. The US National Health and Nutrition Examination Survey 2007 to 2010 results indicate that about 55% of patients with COPD are classified as mild, 40% as moderate, and 5% as severe and very severe.^[24]^ This data is supported by case-finding studies on adults with no previous history of COPD which reveals that most newly diagnosed patients with COPD fall into the categories of mild and moderate.^[25,26]^

## 3. Underdiagnosis and its’ burden

The challenge of delayed diagnosis and underdiagnosis of COPD is indeed substantial, with estimates suggesting that it affects even more than 70% of cases.^[27–33]^ However, despite the considerable evidence, no intervention has been implemented to address this issue. When comparing 3 surveys conducted in Spain over 2 decades, no significant changes were observed in the frequency of underdiagnosis (78%, 73%, and 74%, in 1997, 2007, and 2017, respectively).^[34,35]^ Similar evidence from US National Health and Nutrition Examination Survey indicated underdiagnosis rates of 71% and 72% in 1994 and 2012, respectively.^[36]^

The overall burden of undiagnosed COPD on individuals and the health system is substantial. Underdiagnosis has adverse effect on patients’ quality of life and daily activities.^[37]^ These Undiagnosed patients face a higher risk of all-cause mortality compared to individuals without airway obstructions (hazard ratio, 1.23; 95% CI, 1.08–1.40).^[36]^ While undiagnosed COPD patients experience fewer symptoms and exacerbations than diagnosed COPD patients, they still utilize a similar amount of health services for exacerbation events before receiving a diagnosis.^[38]^

Undiagnosed COPD patients utilized emergency services 1.5 times more often than non-COPD patients. The healthcare costs for undiagnosed patients increase over time, with a notable surge occurring in the month leading up to the COPD diagnosis.^[39]^ The severity and frequency of exacerbation are determinants of healthcare utilization and cost.^[40,41]^ The average cost of managing a moderate exacerbation in Canada in 2006 was $641, which includes outpatient ($126) and emergency services ($515) costs. In the case of a severe exacerbation, the average overall cost was $9557 consisting of outpatient ($114), emergency services ($774), and hospitalization with an average stay of 10 days ($8669).^[42]^ The cost of managing moderate and severe exacerbation in US in 2012 were $269 and $18,120, respectively.^[43]^ In 2016 the average cost per hospitalization for exacerbation in mild, moderate, and severe COPD reached $16,469.^[44]^ The rising cost of emergency visits due to exacerbation underscores the importance of prompt COPD diagnosis to prevent unnecessary healthcare expenditure.

The situation becomes even more alarming when we consider the link between exacerbation and outcome of the disease. The decline in the FEV1 of patients with COPD over time is notably more significant in those experiencing frequent exacerbations compared to those with infrequent exacerbation.^[45]^ Moreover, earlier exacerbations act as strong risk factors for future exacerbations. The odds ratio for experiencing frequent exacerbation reaches 13.6 in patients with 2 or more exacerbations,^[46]^ leading to higher rates of hospitalization accounts for the majority of the costs borne by undiagnosed COPD patients in the year just before the diagnosis.^[47]^

## 4. Early detection: a game-changer for COPD

Previous studies have demonstrated that an early and accurate diagnosis of COPD can yield benefits in both short-term and long-term outcomes. Delaying the diagnosis can postpone the initiation of essential preventive and treatment measures, especially as the disease continues to progress.^[48]^ The pathogenesis pathway leading to COPD begins with inflammatory changes, which are then followed by the destruction of lung parenchymal tissue and the development of airway fibrosis.^[49–51]^ The value of screening activities lies in their capacity to facilitate timely preventive measures and treatments, and this principle holds true for COPD as well. While there exist several medications and preventive interventions aimed at reducing COPD mortality, the crucial first step is accurate diagnosis of the condition. Therefore, establishing a proper diagnosis for COPD is pivotal in addressing the disease and mitigating its impact on individuals’ health and well-being.^[11]^ Early identification of COPD allows for swift interventions, which in turn can significantly enhance patient outcomes and optimize the effectiveness of available treatment options. Providing treatment to patients in the early stages can be especially beneficial, as FEV1 tends to deteriorate at a faster rate during initial phases of COPD.^[52]^

## 5. Root causes of underdiagnosis and delay in diagnosis: patient versus provider versus health system

Multiple factors contribute to the underdiagnosis of COPD, encompassing a range of characteristics, such as younger age, being current or never-smoking or having lower smoking history, lower educational attainment, belonging to racial/ethnic minorities, and notably, mild to moderate airway obstruction. These predictive elements underscore the complexity of COPD diagnosis and emphasize the need for targeted efforts to enhance early detection and comprehensive screening approaches, particularly within populations at a higher risk of underdiagnosis.^[33,36,53]^

On the other hand, the diagnosis of COPD faces several challenges. Some patients experience respiratory symptoms but refrain from seeking medical attention (under-presentation) due to several reasons, including financial constraints, difficulties in adjusting daily routines, limited healthcare access, and finally, the tendency of patients not to report their symptoms until the disease reaches a more advanced stage. COPD symptoms are nonspecific, and many patients, especially those with less severe obstruction, attribute these symptoms to factors such as aging, obesity, smoking, flu, and other exposures.^[54]^ The intermittent nature of symptoms can also contribute to under presentation and subsequent diagnosis at more advanced stages.^[55]^ Over time, patients become accustomed to their symptoms and the frequency of changes, leading to a reduced sense of urgency to report symptoms. In fact, despite physicians’ encouragement for patients with COPD to report exacerbations, only half of them do so.^[56]^ They may not link exacerbations with disease progression, instead perceiving them as temporary obstacles due to environmental changes, infections, or activities.^[21]^ As a result, they tend to rely on self-management strategies and avoid visiting a physician unless they find themselves incapable of managing their symptoms on their own.^[57]^ Given this trend, it is reasonable to assume that under-presentation is more prevalent among individuals who have not yet received a formal diagnosis.

Another substantial challenge in COPD diagnosis pertains to physicians’ awareness and access to appropriate equipment. Some patients do present their symptoms to physicians, but still experience underdiagnosis, primarily due to factors like limited availability of diagnostic tools, lack of awareness among healthcare providers, the overshadowing of COPD by other diseases, and inadequate utilization of diagnostics. Spirometry, the gold standard for diagnosing COPD, is not routinely performed in primary care settings, leading to missed opportunities for early detection. These barriers can hinder timely and accurate identification of COPD, underscoring the importance of enhancing physician education, promoting access to necessary diagnostic tools, and fostering a proactive approach towards COPD screening and diagnosis.^[58–60]^

Addressing these issues through increased awareness, improved screening methods, and enhanced access to spirometry can significantly contribute to reducing the burden of delayed diagnosis and underdiagnosis of COPD.

## 6. Cost-effectiveness of screening and potential saving

Successful screening efforts require addressing significant health issues that involve an asymptomatic phase and available interventions. Sensitivity, specificity, patient acceptance, and cost- effectiveness are crucial for an effective screening method.^[61,62]^ Augusti and colleagues argue that novel strategies must be considered for screening including questionnaires, utilization of micro-spirometers or peak flow meters, built-in microphones on smartphones, or artificial intelligence decision making tools. These methods and their cost-effectiveness are subject to further studies such as ANTES research initiative in Spain.^[63]^ Screening programs often encounter complexities due to the hidden nature of diseases, necessitating a focus on health outcomes rather than solely quantifying new cases. Unlike acute diseases, chronic conditions involve patient adaptation and emotional factors that must not be overlooked.^[64]^ A Chinese modeling study demonstrated that screening for COPD increased patients’ quality-adjusted life years by 0.28 units compared to routine care, with costs below one-tenth of the willingness-to-pay threshold.^[65]^ Active or opportunistic case finding has proved cost-effective in identifying COPD among smokers with respiratory symptoms compared to routine primary care.^[66–68]^ A prescreening approach is shown to decrease the number needed to screen from 8 to 2.1.^[69,70]^ Another modeling study aimed at assessing the cost-effectiveness of care-based case detection in a primary healthcare setting, from the perspective of healthcare payers, considered all Canadian residents aged over 40, irrespective of smoking history. This study evaluated 16 different screening scenarios utilizing the COPD Diagnostic Questionnaire ± spirometry at intervals of 3 to 5 years over a 20-year time horizon. The results of this study suggest that utilizing COPD Diagnostic Questionnaire for individuals over 40 is the most economically efficient intervention. Additionally, all 16 screening scenarios exhibited incremental cost-effectiveness ratios below 50,000 Canadian Dollars per quality-adjusted life years.^[71]^

## 7. Current challenges and opportunities

There are ongoing debates regarding the priority of interventions for COPD management. Some argue that the focus should be on smoking cessation interventions instead of screening for disease caused by smoking, believing that the benefits of screening are overestimated.^[72]^ Others emphasize that screening should result in improved outcomes and management, and if it does not or if there is limited data, a screening program should not be established. Like any health intervention, screening for COPD has both advantages and disadvantages.

Receiving a negative screening result might unintentionally give asymptomatic smokers a reason to continue smoking. nevertheless, taking part in a screening program can also inspire individuals to actively pursue smoking cessation and boost their chances of quitting.^[73]^ For instance, in England, a study involving community pharmacists screened 238 individuals, diagnosing 135 with COPD. Assuming a 33% success rate for smoking cessation, this study projected a cost saving of £ 392.67 per patient.^[74]^ The success rate of smoking cessation is linked to factors such as the degree of airway obstruction and awareness of the obstruction. Individuals with airflow obstruction who are aware of their condition exhibit higher one-year quit rates compared to those without obstruction (15.1% vs. 9.9%, respectively; *P*-value < .05),^[75]^ indicating that awareness of airway obstruction can motivate smoking cessation.

One significant concern is the qualifications required to conduct valid and accurate screening tests in a primary care setting. This includes the need for expert healthcare staff and calibrated equipment, which can be improved through training, tele-medicine, and quality assurance evaluations.^[76–80]^

The COPD assessment in primary care to identify undiagnosed respiratory disease and exacerbation risk is among the initial COPD screening tools that consider the risk of exacerbation and is currently under investigation in the US primary care system.^[81]^ Several other diagnostic tools have been developed to detect clinically significant COPD, and various screening surveys have been launched.^[82–86]^ While these instruments are often user-friendly for patients, their administration should be approached cautiously, considering their limitations. Their moderate accuracy and inability to differentiate between stages of obstruction underscore the necessity of using them in conjunction with spirometry.^[87]^ It is worth noting that many COPD diagnostic questionnaires are validated on smokers and exclude a substantial proportion—often one quarter—of patients with COPD who have never smoked.^[88–90]^ This limitation highlights the need for a more inclusive and comprehensive approach that can accurately identify COPD across various patient groups.

## 8. Suggestion of alternative approach

As mentioned earlier, relying solely on clinical symptoms is not a suitable starting point for the diagnostic approach to COPD due to their lack of sensitivity, specificity, and their inability to compel individuals to seek care. Additionally, symptoms often appear too late in the disease’s progression pathway. However, the challenges and costs associated with utilizing spirometry for every individual must also be acknowledged. Therefore, an alternative approach aimed at quantifying symptoms could be the key to solving this challenge. The COPD assessment test (CAT) score holds promise in this regard, serving as a tool for effective communication between patients and physicians and supplying an objective understanding of the problem and its severity.

Introduced in 2009, CAT is a validated eight-item questionnaire that straightforwardly evaluates the impact of COPD symptoms on patients’ health status,^[91]^ makes it a suitable tool for assessing patients with COPD and facilitating extended follow-up. The CAT score shows a robust correlation with pulmonary function test parameters, both in stable patients and during acute exacerbations. It increases during exacerbations and is associated with decreases in FEV1 and peak expiratory flow (PEF).^[92,93]^ Moreover, there is a correlation between changes in CAT score and future exacerbations.^[94]^ It can be used as a complementary tool to predict exacerbation, health status, depression, and mortality in patients with COPD. However, evidence does not support the equivalence of CAT ≥ 10 and modified Medical Research Council Scale ≥ 2 in categorizing patients into GOLD stages.^[95]^ The CAT total score ranges from zero to 40, with a two-point increase in baseline CAT score being considered clinically significant, indicating a deterioration in health status.^[96]^ Studies have also demonstrated significant correlation between CAT score, breathlessness, and phlegm, and a COPD diagnosis.^[97]^ A pooled study of 18,577 patients with COPD from 41 cohorts revealed that CAT > 18 could serve the same as modified Medical Research Council Scale ≥ 2 in categorizing patients^[98]^; however, for the means of screening and in order to increase the sensitivity of the test and detecting any potential patient, a CAT score of 10 could be an appropriate threshold for requesting spirometry, as fewer than 20 percent of patients with COPD falls within zero to 10 range.^[99]^ Furthermore, administering CAT in primary care is economically efficient and holds value as a necessary part of primary care before other diagnostic procedures are pursued. This approach could aid in streamlining diagnosis while still being cost-effective and relevant to patient needs. It might be advisable for primary care providers to routinely measure the CAT score annually in current smokers who are over 40 years old and have a smoking history of more than 20 pack-years. Spirometry testing could be recommended for individuals who have a high CAT score (10 or above). Further investigation is needed before implementing the CAT score as a tool for COPD screening in primary care settings.

## 9. Conclusion

Screening for COPD plays a crucial role in finding at-risk populations who could potentially benefit from available treatments and interventions. While being symptomatic traditionally serves as the basis for initiating screening, the fact that symptoms have limited accuracy for COPD diagnosis necessitates a rethinking of the concept of being symptomatic. It is imperative to find a way to quantify patients’ perception of their symptoms and facilitate effective communication of this concept.

In this context, utilizing the CAT score as a prescreening tool shows promise. CAT offers validity and reliability as a tool, and its ease of use contributes to its user-friendliness. It can serve as an entry point for screening by providing an objective measure of patients’ symptom experience. The CAT score allows patients and healthcare providers to better understand the impact of symptoms on patients’ health status, facilitating early detection and intervention.

By incorporating CAT scores as part of primary care, the medical community can enhance the accuracy and efficiency of COPD diagnosis, helping to bridge the gap between asymptomatic experiences and effective healthcare interventions. This approach not only addresses the limitations of relying solely on symptoms but also supports a more patient-centered and comprehensive approach to COPD screening, diagnosis, and care.

## Author contributions

**Conceptualization:** Atefeh Vaezi, Mehdi Mirsaeidi.

**Investigation:** Atefeh Vaezi, Mehdi Mirsaeidi.

**Supervision:** Mehdi Mirsaeidi.

**Writing – original draft:** Atefeh Vaezi, Mehdi Mirsaeidi.

**Writing – review & editing:** Atefeh Vaezi, Mehdi Mirsaeidi.
